# 5G Service and Pacemakers/Implantable Defibrillators: What Is the Actual Risk?

**DOI:** 10.3390/ijerph20054512

**Published:** 2023-03-03

**Authors:** Cecilia Vivarelli, Federica Censi, Giovanni Calcagnini, Ermenegildo De Ruvo, Leonardo Calò, Eugenio Mattei

**Affiliations:** 1 Department of Cardiovascular, Endocrine-Metabolic Diseases and Aging, Italian National Institute of Health, 00161 Rome, Italy; 2 Department Ingegneria Civile e Ingegneria Informatica (DICII), University of Rome Tor Vergata, 00133 Rome, Italy; 3 Policlinico Casilino, 00169 Rome, Italy

**Keywords:** 5G, EMI, pacemakers, ICDs, exposure assessment

## Abstract

Rapidly growing worldwide, 5G service is expected to deeply change the way we communicate, connect and share data. It encompasses the whole spectrum of new technology, infrastructure and mobile connectivity, and will touch not only every sector in the industry, but also many aspects of our everyday life. Although the compliance with international regulations provides reasonable protection to public health and safety, there might be specific issues not fully covered by the current technical standards. Among the aspects that shall be carefully considered, there is the potential interference that can be induced on medical devices, and in particular on implantable medical devices that are critical for the patient’s life, such as pacemakers and implantable defibrillators. This study aims to assess the actual risk that 5G communication systems pose to pacemakers and implantable defibrillators. The setup proposed by the ISO 14117 standard was adapted to include 5G characteristic frequencies of 700 MHz and 3.6 GHz. A total number of 384 tests were conducted. Among them, 43 EMI events were observed. Collected results reveal that RF hand-held transmitters operating in these two frequency bands do not pose additional risk compared to pre-5G bands and that the safety distance of 15 cm typically indicted by the PM/ICD manufacturer is still able to guarantee the patient’s safety.

## 1. Introduction

### 1.1. Technology for 5G

The technology for 5G aims at being much more than a simple evolution of 4G, as it will be a key enabler of the future digital world. It has been designed to meet the extensive growth in data and connectivity that characterizes today’s modern society, just as previous generation technologies have supported economic growth and development in the past, and it is expected to become a dominant General Purpose Technology in the coming decade [1].

Compared to pre-5G technology, 5G sites do not need a higher power rating, whereas they will increase the data transfer speed, thanks both to a more efficient use of frequency bands leveraging complex signal processing and to the use of frequencies higher than those utilized in current cellular networks. In particular, 5G technology is designed to operate in two distinct frequency ranges (FR) [2]:

- FR1, from 410 MHz up to 7.125 GHz;
- FR2, from 24.250 GHz up to 52.600 GHz.

Part of the FR1 band was already used in LTE and pre-5G technology (LTE ranges from 600 MHz up to 6 GHz), but now new frequency bands have been specifically dedicated to 5G signals. This band is useful for guaranteeing an optimal balance between coverage and capacity for both rural and urban areas. On the other hand, FR2 band (usually referred as millimeter wave band) is the newest one, only implemented by the 5G communication system, and it is supposed to support a smaller coverage area. Since the relatively lower power and EIRP compared to the sub-6 GHz systems, these base stations will be installed close to users.

Among all those frequency bands, the ones that are today actually deployed for 5G services by mobile service providers are those whose central frequency is 3.6 GHz and 700 MHz. However, in both these bands, mobile service providers are currently deploying the 5G NSA (Non-Standalone) standard, mostly relying on LTE infrastructure.

Similar to what happened with the pre-5G technologies, the new 5G deployments will have to ensure the same level of safety in mobile communications, and already existing RF-EMF standards and recommendations for existing frequency must take into account the peculiar elements that 5G systems have introduced. The compliance with international regulations provides reasonable protection of the general population against possible adverse effects.

Indeed, as any new technology, there might be specific issues not fully covered by the current technical standards. The technology for 5G will use different antennas, frequencies and infrastructures from pre-5G systems. In the last years, many concerns about the possible consequences of 5G systems on human health have been raised in the general population, often not based on any scientific evidence. Thus, it is worthwhile to provide evidence-based data and collect experimental measurements to assess if 5G systems pose actual risks for human health. One aspect that may need further investigation is the possible interference of these new generation sources with medical devices, in particular with active implantable medical devices (AIMDs), such as pacemakers and implantable cardiac defibrillators (ICDs). 

### 1.2. AIMD

Pacemakers and defibrillators are AIMDs intended to treat cardiac arrhythmias [3,4]. These devices have an electronic component that “senses” the heart activity and generates electrical impulses when needed. Typically, the ability to sense the electrical activity of the heart and to transmit electrical impulses to the heart is guaranteed through electrical connections called leads, which are positioned inside the heart. Currently there are also leadless pacemakers, recently introduced on the market, which are small in size and which are inserted and fixed directly into the heart. In addition, among the ICDs, the subcutaneous ICD has the lead wire tunneled underneath the skin next to the sternum. Over one million cardiac pacemakers [5] and over 200,000 implantable defibrillators [6] are implanted every year worldwide, and these numbers are expected to grow given the aging population and increasing clinical indications.

The number and the types of electromagnetic (EM) emitters to which patients with AIMD are exposed in their day-to-day activities has proliferated over the past two decades. This trend is continuously evolving. The interaction between these emitters and AIMD is an ongoing concern of patients, industry and regulators, given the potential life-sustaining nature of these devices. The risks associated with such interactions include device inhibition or delivery of inappropriate therapy that, in the worst case, could result in serious injury or patient death.

### 1.3. Electromagnetic Immunity of AIMD

Regulatory requirements, demanded to manufacturers prior to placing devices on the European market, are based on the assumption that devices’ proper functioning must be assured even when they are exposed to electromagnetic fields up to the ICNIRP General Public Reference Levels [7,8,9,10,11,12,13,14].

Standard test methodologies allow manufacturers to evaluate the EM compatibility performance of a product and demonstrate that the product achieves an appropriate level of EM compatibility in uncontrolled EM environments that patients might encounter. The standard ISO 14117:2019 active implantable medical devices—electromagnetic compatibility [7] defines, in the frequency range from 385 MHz to 3 GHz, a set of radiated tests at selected frequencies: 385, 450, 600, 800, 825, 850, 875, 900, 930, 1610, 1850, 1910, 2450 and 3000 MHz. These frequencies are not equally spaced on the spectrum. Indeed, they have been chosen as they are the working frequencies of the most common hand-held communication devices that are typically used in our everyday life (e.g., wireless telephones). 

The upper-frequency limit of 3 GHz chosen in the ISO standard reflects consideration of the following factors: (i) the limited types of radiators of frequencies above 3 GHz; (ii) the increased device protection afforded by the attenuation of the enclosure and body tissue at microwave frequencies; (iii) the expected performance of EMI control features that typically are implemented to meet EMI requirements at low frequencies; (iv) the reduced sensitivity of circuits at microwave frequencies.

However, emitters’ technology is updating faster than international standards and 5G is one of the most dramatic examples: the ISO 14117 does not take into account 5G central band frequencies in its tests, such as 700 MHz and 3.6 GHz. Although there is the already mentioned rationale that explained why frequencies above 3 GHz are not tested, this choice was made by looking only to pre-5G technology, where these frequencies were not used. Today, 5G terminals have become largely used by the general population and are expected to continue their growth in the next years. Thus, it is worthy to extend the EMI tests above 3 GHz. In addition, 700 MHz is expected to become one of the most exploited frequencies of FR1 in 5G communication systems, and it is not included among the frequencies to be tested according to [7]. 

The aim of this study is to investigate, through in vitro testing on commercial devices, whether these two bands (700 MHz and 3.6 GHz) of 5G systems could pose an additional risk, in terms of electromagnetic interference, for PMs and ICDs. More specifically, the setup proposed by the ISO 14117 standard has been adapted to include the two frequencies of interest and provocative testing has been performed. RF power levels higher that those indicated in the standard have been adopted, not only to investigate any potential EMI phenomena, but also to estimate the safety margin with respect to the actual power typically used by commercial 5G terminals. 

## 2. Materials and Methods

Six PMs and four ICDs from five of the most important international manufacturers (Abbot, Biotronik, Boston, Medtronic, Microport) were tested. The devices were explanted from patients at the “Policlinico Casilino Hospital” (Rome, Italy) between October and November 2022, due to the ERI (elective replacement indication) alarm activation and can be considered a representative sample of what is today implanted in the population. The list of the devices, together with the specific model’s name and programmed parameters are reported in Table 1 (PMs) and Table 2 (ICDs). 

PMs were programmed in single chamber mode (AAI or VVI), unipolar pacing, at the maximum sensitivity allowed by the manufacturer. When possible, PMs were tested both in unipolar and in bipolar sensing mode. When programmed with unipolar sensing, both unipolar and bipolar leads were tested. 

ICDs were programmed in bipolar mode both for pacing and sensing. For each ICD, two detection windows were activated: in the first window (from 150 to 200 beats per minute–bpm), the detection of ventricular tachycardia (VT) was activated and the delivery of the antitachycardia pacing (ATP) therapy was programmed at the most sensible conditions. In the second window (bpm > 200), the detection of VF was activated, and the delivery of high-voltage shocks was programmed at the at the most sensible conditions allowed by each manufacturer.

### 2.1. Test Setup

**Figure 1:**
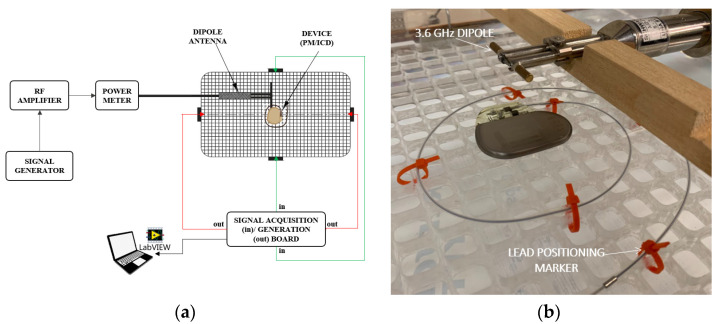
(**a**) Schematic representation of the test setup according to ISO 14117. (**b**) Highlight of one test conducted on a PM.

The test setup was designed on the basis of what is described in the ISO 14117 standard [7]. It consists of the following (Figure 1a):

1. The torso simulator: a PVC transparent tank (60 × 40 × 15 cm^3^, volume ~28.6 L) filled with saline solution having a conductivity 0.27 S/m. A PVC grid (20 × 38 cm^2^) was placed in the central area of the tank, to support the device under test (DUT) and allow for a stable and reproducible arrangement of the leads. The height of the grid was adjusted to have a distance of 0.5 cm between the top surface of the DUT and the saline solution. The PM/ICD lead was configured in a spiral, as indicated by the ISO 14117 standard in the test requirements section for the frequency range of 385 MHz ≤ ƒ ≤ 3 GHz. Several markers were positioned on the grid to facilitate the positioning of the lead (Figure 1b) and to guarantee reproducible conditions among all the measurements.

Two pairs of stainless-steel electrode plates placed on the walls of the tank were used to monitor and test the device while it was immersed in the saline. Each plate measured 5 cm × 5 cm × 0.2 cm and was positioned at the middle of one of the inner walls of the torso simulator. One pair of plates was placed on opposite walls of the torso simulator and it allows the monitoring of the DUT. The second pair of plates was placed on the other opposite walls and it allows ECG simulation signals to be applied to the device leads through the saline. An imaginary line connecting one pair of plates is perpendicular to the imaginary line connecting the other pair of plates. This minimized the cross-talk between the injection and monitoring plates. The plates were connected to a custom-made circuit and to an A/D converted USB card (USB 6240, National Instruments, Austin, TX, USA) driven by a PC, which records the signal received from one pair of plates and generates the simulated ECG applied to the other pair of plates. A custom-made graphic user interface developed in LabVIEW (National Instruments, Austin, TX, USA) was used to monitor in real time the PM/ICD behavior during the test, to turn on/off the simulated ECG and to store on the PC memory the recordings acquired from the torso simulator.

2. The RF generation and monitoring system: the RF signal was generated by two dipole antennas, operating at 700 MHz and 3.6 GHz, respectively. Before starting the tests, the antennas were tuned to have an SWR (standing wave ratio) < 1.5 at the frequencies of interest, when operating in free space. The antennas were placed upon the torso simulator, at a height of 2 cm from the saline solution (2.5 cm from the top surface of the device under test). A wooden bracket was used to support the antenna and to maintain the desired separation distance from the saline solution. An RF signal generator (SMT03, Rohde & Schwarz, Munich, Germany) and a 50W power amplifier (ZHL-50W-63+, Minicircuit, New York, NY, USA) were used to feed the dipole antennas. An in-line RF power meter (NRT-Z44, Rohde & Schwarz, Germany) was used during all the tests to monitor the SWR value and the actual net power (forward–reverse) delivered to the torso simulator. The whole system was able to reach a net power up to 40 W at 700 MHz and up to 20 W at 3.6 GHz.

### 2.2. Test Procedures—PM

For each PM, two tests were performed:

Test 1—Simulated ECG signal off: with the simulated ECG off and the RF signal off, the PM activity was recorded for 5 s. Then, the RF signal was switched on at the maximum power allowed by the RF generation system (i.e., 40 W at 700 MHz and 20 W at 3.6 GHz). The activity of the PM was monitored for 10 s. If EMI occurred, the net power into the antenna was decreased until the EMI phenomenon ceased. 

Test 2—Simulated ECG signal on: with the simulated ECG off and the RF signal off, the PM activity was recorded for 5 s. Then, the simulated ECG was turned on, at a frequency 10% greater than the programmed pacing rate of the DUT. The amplitude of the ECG signal was raised from zero to a point where the DUT tracks the signal, and then the amplitude of the signal was doubled to ensure sufficient sensing. Afterwards, the RF signal was switched on at the maximum power allowed by the RF generation system (>40 W), and activity of the PM was monitored for 10 s. If EMI occurred, the net power into the antenna was decreased until the EMI phenomenon ceased.

The two tests were conducted with the RF signal generated continuously and pulse-modulated at 500 ms intervals, signal on for 25 ms, as prescribed by the ISO 14117 standard. This modulation reproduces the worst-case condition able to induce the erroneous pacing inhibition. Indeed, the repetition frequency of 2 Hz is equivalent to a sinus rhythm of 120 bpm, that is likely to be interpreted by the PM as a spontaneous rhythm and to cause pacing inhibition. 

The tests were repeated twice, changing the position of the dipole antenna:

- Position 1: antenna parallel to the major side of the torso simulator, thus dipole perpendicular to PM connector;
- Position 2: antenna parallel to the minor side of the torso simulator, thus dipole parallel to PM connector;

Each PM was tested according to the procedure described above in two sensing modalities (unipolar and bipolar). When programmed in unipolar mode, both unipolar and bipolar leads were considered. 

**Figure 2:**
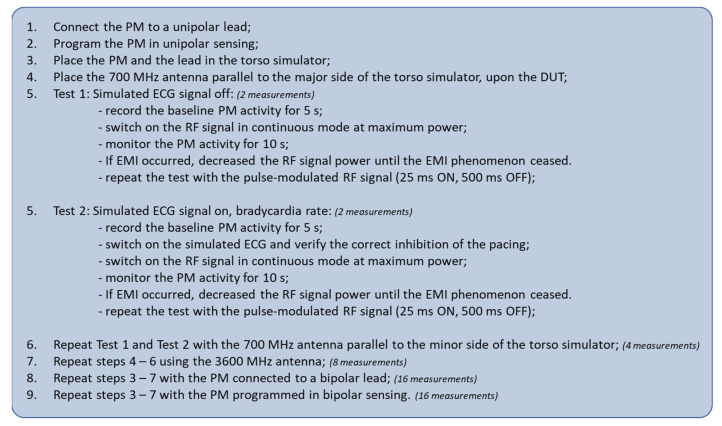
The flow chart of the test procedure followed for PM measurements. A total of 48 measurements for each PM were collected.

The flow chart of the test procedure followed for PMs measurements is reported in Figure 2. A total of 48 measurements for each PM were collected.

### 2.3. Test Procedures—ICD

For each ICD, three tests were performed:

Test 1—Simulated ECG signal off: with the simulated ECG off and the RF signal off, the ICD activity was recorded for 5 s. Then, the RF signal was switched on at the maximum power allowed by the RF generation system (i.e., 40 W at 700 MHz and 20 W at 3600 MHz). The activity of the ICD was monitored for 15 s. If EMI occurred, the net power into the antenna was decreased until the EMI phenomenon ceased. 

Test 2—Simulated ECG signal on, bradycardia rate: with the simulated ECG off and the RF signal off, the PM activity was recorded for 5 s. Then, the simulated ECG was turned on, at a frequency 10% greater than the programmed pacing rate of the DUT. The amplitude of the simulated ECG signal was raised from zero to a point where the DUT tracks the signal, and then the amplitude of the signal was doubled to ensure sufficient sensing. Afterwards, the RF signal was switched on at the maximum power allowed by the RF generation system, and activity of the ICD was monitored for 15 s. If EMI occurred, the net power into the antenna was decreased until the EMI phenomenon ceased.

Test 3—Simulated ECG signal on, tachycardia rate: with the simulated ECG off and the RF signal off, the PM activity was recorded for 5 s. Then, the RF signal was switched on at the maximum power allowed by the RF generation system, together with the simulated ECG, at a frequency rate within the programmed tachycardia detection window of the DUT (160 bpm for all the ICD tested). The amplitude of the simulated ECG at tachycardia rate was the same adopted for the simulated ECG at bradycardia rate (Test 2). The activity of the ICD was monitored for 15 s. If EMI occurred, the net power into the antenna was decreased until the EMI phenomenon ceased.

The three tests were conducted with the RF signal generated continuously and pulse-modulated (modulation A—25 ms ON and 475 ms OFF). In Test 1 and 3, the ICDs were additionally tested with a pulse-modulated RF signal having a shorter repetition time (modulation B—25 ms ON and 330 ms OFF), resulting in an interference signal with a repetition frequency of about 2.8 Hz, which is equivalent to a tachycardia of about 170 bpm. Such a rhythm was empirically chosen to fall within the programmed tachycardia detection window of the ICDs tested.

As for PM measurements, the tests were repeated twice, changing the position of the dipole antenna:

- Position 1: antenna parallel to the major side of the torso simulator, thus dipole perpendicular to PM connector;
- Position 2: antenna parallel to the minor side of the torso simulator, thus dipole parallel to PM connector.

Only the bipolar sensing modality was tested, as the unipolar one is not allowed by the ICD manufacturers for the devices today present on the market. 

**Figure 3:**
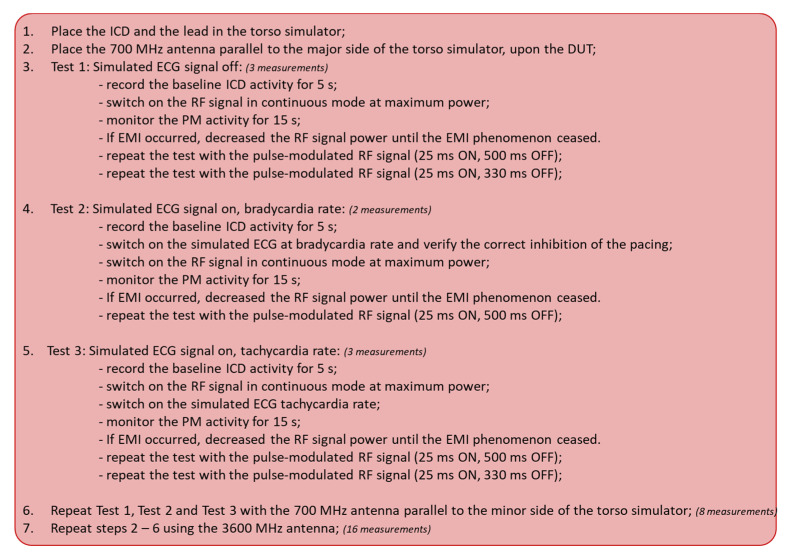
The flow chart of the test procedure followed for ICDs measurements. A total of 32 measurements for each ICD were collected.

The flow chart of the test procedure followed for ICD measurements is reported in Figure 3. A total of 32 measurements for each ICD were collected.

### 2.4. Performance Criteria—PM

Test 1—Simulated ECG signal off: an EMI event was considered to occur if the PM exhibited any deviation in pace-to-pace interval that exceeded 10% of the programmed rate.

Test 2—Simulated ECG signal on: an EMI event was considered to occur if the PM exhibited any pace pulse during application of the simulated ECG and RF signals.

### 2.5. Performance Criteria—ICD

Test 1—Simulated ECG signal off: an EMI event was considered to occur if the ICD exhibited either of the following characteristics:

- Any deviation in pace-to-pace interval that exceeded 10% of the programmed rate;
- Delivery of defibrillation or cardioversion pulse to the high-voltage electrodes;
- Delivery of antitachycardia pacing (ATP) to the pacing leads.

Test 2—Simulated ECG signal on, bradycardia rate: an EMI event was considered to occur if the ICD did not exhibit any pace pulse during application of the simulated ECG and RF signals.

Test 3—Simulated ECG signal on, tachycardia rate: an EMI event was considered to occur if the ICD did not deliver the appropriate ATP therapy, according to the programmed parameters.

## 3. Results

We conducted a total of 384 tests (256 for PMs and 128 for ICDs) and the results for each device are reported in Table 3 (PMs—unipolar sensing), Table 4 (PMs—bipolar sensing) and Table 5 (ICDs). We assigned a random number to identify each device, reported in the first column of these tables.

None of tested devices, both for PMs and ICDs, showed EMI events or anomalies when using the RF signal at 3.6 GHz. We observed 43 EMI events at 700 MHz.

A color scale legend was used to represent the severity of the observed EMI event, as a function of the power of the RF interfering signal:

- Red: the EMI event was observed under the threshold of 120 mW net power into the dipole antenna, that is the minimum power level at which the devices shall not exhibit any degradation of its performances according to the ISO 14117 standard (test section for the frequency range of 385 MHz ≤ ƒ ≤ 3 GHz);
- Orange: the EMI event occurred in the power range from 120 mW to 2 W (for tests at 3.6 GHz) or from 120 mW to 8 W (for tests at 700 MHz). 2 W and 8 W are the power levels for the optional EMI characterization than manufacturer can perform according to the ISO 14117 standard, at frequencies below and over 1 GHz, respectively.
- Yellow: the EMI event occurred for power levels greater than 2 W/8 W, for measurements at 700 MHz/3.6 GHZ, respectively.
- Green: no EMI events were observed.

Power levels reported in tables are the power threshold levels at which interference is still observed; below that value, PM/ICD returns to its programmed functioning.

**Figure 4:**
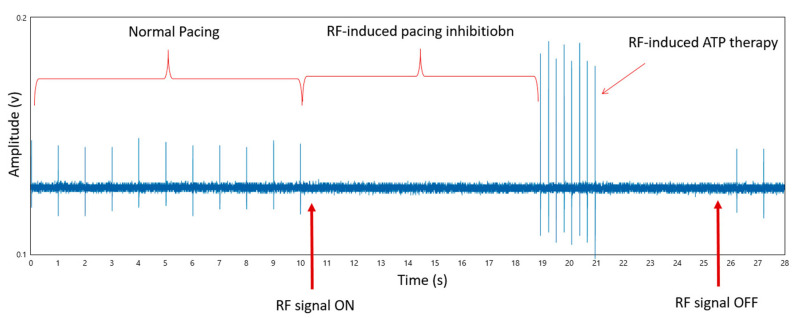
Example of acquired signal on an ICD recording an EMI event.

An example of an EMI event recorded during the tests is reported in Figure 4. 

### 3.1. Pacemakers

None of the PMs tested showed EMI events with a net power into a dipole antenna below 120 mW.

- Test 1—Simulated ECG signal off: all the EMI events occurred during this test (pacing test). The RF signal caused the inappropriate PM inhibition. Moreover, EMI was almost exclusively triggered by the pulse modulated RF signal. The EMI even was observed only in one PM (#1), programmed with bipolar sensing, and also with the continuous RF signal. Besides EMI events, other types of minor anomalies (with no clinical significance) were collected: in particular, the missing of a single pulse when the RF pulsed signal was switched on/off.
- Test 2—Simulated ECG signal on: no EMI event was observed in this test (sensing test), in any of the measurement conditions. Just a minor anomaly was recorded for a PM (#6) with unipolar sensing, for power level above 40 W: the RF sinusoidal signal was able to trigger the delivery of unexpected, isolated pulses.

#### 3.1.1. Dipole Positioning 

With PMs programmed with unipolar sensing (Table 3), the dipole position parallel to PM connector (position number 2) is more likely to cause EMI events, especially with unipolar lead: this specific position triggered EMI events below optional power threshold in almost all PMs (#1,2,3,4,6), whilst the dipole position perpendicular to PM connector (position number 1) was less able to cause EMI events, which were recorded, below threshold, only in three PMs (#1,2,6). On the contrary, PMs programmed with bipolar sensing (Table 4) are mostly affected by dipole position number 1, causing EMI in all of the tested PMs (below threshold in #1,3 and above threshold in #2,4); instead, dipole position number 2 triggered an EMI event only in one PM (#1).

**Table 3:** PM results, unipolar sensing (power threshold levels are reported rounded to the nearest integer in terms of Watts).

PM	MODE	SENSITIVITY [MV]	LEAD	PULSED MODE	TEST 1 SIMULATED ECG SIGNAL OFF				TEST 2 SIMULATED ECG SIGNAL ON, BRADYCARDIA RATE			
					700 MHZ		3.6 GHZ		700 MHZ		3.6 GHZ	
					ANT. 1	ANT. 2	ANT. 1	ANT. 2	ANT. 1	ANT. 2	ANT. 1	ANT. 2
1	AAI	0.5	BIPOLAR	OFF	NO EMI	NO EMI	NO EMI	NO EMI	NO EMI	NO EMI	NO EMI	NO EMI
			BIPOLAR	ON	ABOVE 2 W	ABOVE 2 W	NO EMI	NO EMI	NO EMI	NO EMI	NO EMI	NO EMI
			UNIPOLAR	OFF	NO EMI *	NO EMI	NO EMI	NO EMI	NO EMI	NO EMI	NO EMI	NO EMI
			UNIPOLAR	ON	ABOVE 1 W	ABOVE 1 W	NO EMI	NO EMI	NO EMI	NO EMI	NO EMI	NO EMI
2	VVI	1	BIPOLAR	OFF	NO EMI	NO EMI	NO EMI	NO EMI	NO EMI	NO EMI	NO EMI	NO EMI
			BIPOLAR	ON	ABOVE 10 W	ABOVE 8 W	NO EMI	NO EMI	NO EMI	NO EMI	NO EMI	NO EMI
			UNIPOLAR	OFF	NO EMI *	NO EMI *	NO EMI	NO EMI	NO EMI	NO EMI	NO EMI	NO EMI
			UNIPOLAR	ON	ABOVE 6 W	ABOVE 4 W	NO EMI	NO EMI	NO EMI	NO EMI	NO EMI	NO EMI
3	AAI	0.25	BIPOLAR	OFF	NO EMI	NO EMI	NO EMI	NO EMI	NO EMI	NO EMI	NO EMI	NO EMI
			BIPOLAR	ON	ABOVE 8 W	ABOVE 7 W	NO EMI	NO EMI	NO EMI	NO EMI	NO EMI	NO EMI
			UNIPOLAR	OFF	NO EMI	NO EMI *	NO EMI	NO EMI	NO EMI	NO EMI	NO EMI	NO EMI
			UNIPOLAR	ON	ABOVE 19 W	ABOVE 7 W	NO EMI	NO EMI	NO EMI	NO EMI	NO EMI	NO EMI
4	SSI	0.25	BIPOLAR	OFF	NO EMI	NO EMI	NO EMI	NO EMI	NO EMI	NO EMI	NO EMI	NO EMI
			BIPOLAR	ON	ABOVE 10 W	ABOVE 10 W	NO EMI	NO EMI	NO EMI	NO EMI	NO EMI	NO EMI
			UNIPOLAR	OFF	NO EMI	NO EMI	NO EMI	NO EMI	NO EMI	NO EMI	NO EMI	NO EMI
			UNIPOLAR	ON	ABOVE 9 W	ABOVE 7 W	NO EMI	NO EMI	NO EMI	NO EMI	NO EMI	NO EMI
5	VVI	1	BIPOLAR	OFF	NO EMI	NO EMI	NO EMI	NO EMI	NO EMI	NO EMI	NO EMI	NO EMI
			BIPOLAR	ON	ABOVE 40 W	ABOVE 38 W	NO EMI	NO EMI	NO EMI	NO EMI	NO EMI	NO EMI
			UNIPOLAR	OFF	NO EMI	NO EMI	NO EMI	NO EMI	NO EMI	NO EMI	NO EMI	NO EMI
			UNIPOLAR	ON	ABOVE 10 W	ABOVE 8 W	NO EMI	NO EMI	NO EMI	NO EMI	NO EMI	NO EMI
6	AAI	0.4	BIPOLAR	OFF	NO EMI	NO EMI	NO EMI	NO EMI	NO EMI **	NO EMI	NO EMI	NO EMI
			BIPOLAR	ON	ABOVE 7 W	ABOVE 4 W	NO EMI	NO EMI	NO EMI	NO EMI	NO EMI	NO EMI
			UNIPOLAR	OFF	NO EMI	NO EMI	NO EMI	NO EMI	NO EMI **	NO EMI	NO EMI	NO EMI
			UNIPOLAR	ON	ABOVE 3 W	ABOVE 2 W	NO EMI	NO EMI	NO EMI	NO EMI	NO EMI	NO EMI
					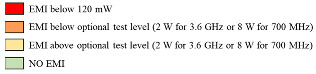							

* One pulse missed when the RF signal is turned on/off. ** Single pulse delivered. EMI: pacing inhibition.

**Table 4:** PM results, bipolar sensing (power threshold levels are reported rounded to the nearest integer in terms of Watts).

PM	MODE	SENSITIVITY [mV]	LEAD	PULSED MODE	TEST 1 SIMULATED ECG SIGNAL OFF				TEST 2 SIMULATED ECG SIGNAL ON, BRADYCARDIA RATE			
					700 MHZ		3.6 GHZ		700 MHZ		3.6 GHZ	
					ANT. 1	ANT. 2	ANT. 1	ANT. 2	ANT. 1	ANT. 2	ANT. 1	ANT. 2
1	AAI	0.1	BIPOLAR	OFF	ABOVE 15 W	NO EMI *	NO EMI	NO EMI	NO EMI	NO EMI	NO EMI	NO EMI
			BIPOLAR	ON	ABOVE 4 W	ABOVE 5 W	NO EMI	NO EMI	NO EMI	NO EMI	NO EMI	NO EMI
2	VVI	1	BIPOLAR	OFF	NO EMI	NO EMI	NO EMI	NO EMI	NO EMI	NO EMI	NO EMI	NO EMI
			BIPOLAR	ON	ABOVE 40 W	NO EMI	NO EMI	NO EMI	NO EMI	NO EMI	NO EMI	NO EMI
3	AAI	0.1	BIPOLAR	OFF	NO EMI	NO EMI	NO EMI	NO EMI	NO EMI	NO EMI	NO EMI	NO EMI
			BIPOLAR	ON	ABOVE 7 W	NO EMI	NO EMI	NO EMI	NO EMI	NO EMI	NO EMI	NO EMI
4	SSI	0.25	BIPOLAR	OFF	NO EMI	NO EMI	NO EMI	NO EMI	NO EMI	NO EMI	NO EMI	NO EMI
			BIPOLAR	ON	ABOVE 19 W	NO EMI	NO EMI	NO EMI	NO EMI	NO EMI	NO EMI	NO EMI
					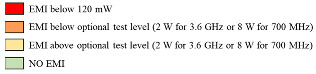							

* One pulse missed when the RF signal is turned on/off. EMI: pacing inhibition.

#### 3.1.2. Sensing Modality

Results on PMs programmed with unipolar and bipolar sensing did not show significant differences between the two: threshold power levels are generally higher for PMs programmed with bipolar sensing, but such difference is marked only for two PMs, whereas they are comparable for the other the power levels that caused EMI events.

#### 3.1.3. Lead Effect

In PMs programmed with unipolar sensing, both unipolar and bipolar leads were tested. Unipolar leads resulted in being more susceptible to EMI events than the bipolar leads; the bipolar lead was capable of inducing an EMI event at the same or lower power level than the unipolar lead only in PM #4.

### 3.2. ICDs

None of the ICDs tested showed EMI events with a net power into a dipole antenna below 120 mW.

One ICD resulted in being completely immune to the RF signal even at the higher power levels reached during the tests. The results are summarized in Table 5.

Compared to PMs, where a single type of EMI was observed, ICDs showed two types of EMI events: the device inhibition and the inappropriate therapy delivery (ATP and/or high voltage shock). Whenever different types of EMI events were observed in a single test, the power level, as reported in Table 5, corresponded to the lowest power level at which the first EMI events was observed. In particular, the EMI measurements showed:

- Test 1—Simulated ECG signal off: three ICDs (#1, 2, 3) were inhibited by the RF-pulsed signal (modulation A) at 700 MHz. Moreover, the RF-pulsed signal (modulation B) also triggered the inappropriate delivering of therapy (ATP ICD #2,3, high shock voltage ICD # 1). Such EMI events were observed with the same threshold power levels observed during the tests using modulation A. Only ICD #1 exhibited inappropriate therapy delivered (high voltage shock) at a greater threshold (25 W).
- Test 2—Simulated ECG signal on, bradycardia rate: no EMI event was observed during this test, meaning that once the device is properly inhibited due to the delivery of a simulated ECG signal with 70 bpm, it successfully remains in this state despite all different RF interfering signals being employed.
- Test 3—Simulated ECG signal on, tachycardia rate: ICDs # 2 and 3 showed an EMI event in the form of inappropriate therapy delivery, consequently to RF-pulsed signal interference.

For ICD, only the bipolar sensing modality was allowed. As far as antenna positioning is concerned, dipole position parallel to ICD connector (position number 2) seemed to represent a preferential coupling factor for an EMI event to occur. The opposite phenomenon happened only in ICD #1.

**Table 5:** ICD results, bipolar sensing (power threshold levels are reported rounded to the nearest integer in terms of Watts).

ICD	SENSITIVITY [mV]	VT VF [min^−1^]	LEAD	PULSED MODE	TEST 1 SIMULATED ECG SIGNAL OFF				TEST 2 SIMULATED ECG SIGNAL ON, BRADYCARDIA RATE				TEST 3 SIMULATED ECG SIGNAL ON, TACHICARDIA RATE			
					700 MHZ		3.6 GHZ		700 MHZ		3.6 GHZ		700 MHZ		3.6 GHZ	
					ANT. 1	ANT. 2	ANT. 1	ANT. 2	ANT. 1	ANT. 2	ANT. 1	ANT. 2	ANT. 1	ANT. 2	ANT. 1	ANT. 2
1	A: 0.25 V: 0.15	VT: 150–200 VF > 200	DF-1	OFF	NO EMI *	NO EMI	NO EMI	NO EMI	NO EMI	NO EMI	NO EMI	NO EMI	NO EMI	NO EMI	NO EMI	NO EMI
				ON 1	ABOVE 3 W	ABOVE 5 W	NO EMI	NO EMI	NO EMI	NO EMI	NO EMI	NO EMI	NO EMI	NO EMI	NO EMI	NO EMI
				ON 2	ABOVE 3 W	ABOVE 9 W	NO EMI	NO EMI	NO EMI	NO EMI	NO EMI	NO EMI	NO EMI	NO EMI	NO EMI	NO EMI
2	AUTO	VT: 157–200 VF > 200	DF4	OFF	NO EMI	NO EMI	NO EMI	NO EMI	NO EMI	NO EMI	NO EMI	NO EMI	NO EMI	NO EMI	NO EMI	NO EMI
				ON 1	NO EMI	ABOVE 25 W	NO EMI	NO EMI	NO EMI	NO EMI	NO EMI	NO EMI	NO EMI	ABOVE 25 W	NO EMI	NO EMI
				ON 2	NO EMI	ABOVE 25 W	NO EMI	NO EMI	NO EMI	NO EMI	NO EMI	NO EMI	NO EMI	NO EMI	NO EMI	NO EMI
3	A: 0.15 V: 0.15	VT: 158–207 VF > 200	DF4	OFF	NO EMI *	NO EMI*	NO EMI	NO EMI	NO EMI	NO EMI	NO EMI	NO EMI	NO EMI	NO EMI	NO EMI	NO EMI
				ON 1	ABOVE 12W	ABOVE 3 W	NO EMI	NO EMI	NO EMI	NO EMI	NO EMI	NO EMI	ABOVE 12 W	ABOVE 3 W	NO EMI	NO EMI
				ON 2	ABOVE 12 W	ABOVE 3 W	NO EMI	NO EMI	NO EMI	NO EMI	NO EMI	NO EMI	NO EMI	NO EMI	NO EMI	NO EMI
4	AUTO	VT: 154–250 VF > 250	DF4	OFF	NO EMI	NO EMI	NO EMI	NO EMI	NO EMI	NO EMI	NO EMI	NO EMI	NO EMI	NO EMI	NO EMI	NO EMI
				ON	NO EMI	NO EMI	NO EMI	NO EMI	NO EMI	NO EMI	NO EMI	NO EMI	NO EMI	NO EMI	NO EMI	NO EMI
				ON	NO EMI	NO EMI	NO EMI	NO EMI	NO EMI	NO EMI	NO EMI	NO EMI	NO EMI	NO EMI	NO EMI	NO EMI
					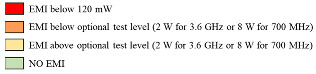											

* One pulse missed when the RF signal is turned on/off. EMI: pacing inhibition; ATP delivering, high voltage shock.

## 4. Discussion

The peculiar innovations that 5G technology introduces compared to pre-5G systems implies a careful evaluation of the impact on safety. In this study, the potential risks for patients with AIMD, in particular PM and ICD, were investigated. Since 5G terminals do not need a power rating higher than pre-5G systems, the main difference on which we focused was the new frequency bands adopted. The setup proposed by the ISO 14117 standard was adapted to include the frequencies of 700 MHz and 3.6 GHz, which are not specifically tested in the standard.

According to [7], for frequencies of 385 MHz ≤ ƒ ≤ 3 GHz, a radiated near field test method is required. The objective of the near field test is to approximate the exposure of portable transmitters in proximity to the DUT. The 120 mW net power level prescribed in ISO 14117 [7] allows a high level of confidence for which an implantable DUT will not be affected by EMI from a hand-held emitter at a distance of 15 cm. A manufacturer can perform the optional characterization tests to provide a reasonable assurance of uninfluenced function of the DUT at close distances (2 cm or less) from a hand-held transmitter. These optional characterization tests require dipole net power levels of 8 W in the frequency range 385 MHz ≤ ƒ < 1 GHz and 2 W in the frequency range 1 GHz ≤ ƒ ≤ 3 GHz. These power levels were selected on the basis of the maximum power levels likely to be encountered from the portable and hand-held radios and cellular telephones on the market at the time the ISO 14117 was written and they can be considered still valid for new 5G terminals. On the other hand, the new frequency bands are not considered in the standard: tests are limited up to 3 GHz, and the closest frequencies to 700 MHz are 600 MHz and 800 MHz. It is important to highlight that, in the band typically used by pre 5G-system (i.e., 800–900 MHz), the ISO standard identifies five frequency tests, with a span of 25 MHz.

It was not possible to test all of the six explanted PMs with bipolar sensing mode because in two of them, the ERI state did not allow for a sensing other than unipolar.

The first important result is that RF signals at 3.6 GHz do not trigger EMI events for net power levels up to 40 W, thus ensuring a solid immunity of PMs and ICDs. As already mentioned, this fact is probably due to various factors, such as the skin-depth effect, the reduced sensitivity of circuits at microwave frequencies and the expected performance of EMI control features. As far as 700 MHz is concerned, all of the tested devices showed an unperturbed behavior up to 120 mW, which is the immunity level that PMs/ICDs must comply with according to [7]. There is only one device that is completely immune up to the maximum tested power (40 W). In all the conducted tests (384), a total number of 43 EMI events were observed. Among them, 22 EMI events occurred under the optional power level threshold (2 W at 3.6 GHz or 8 W at 700 MHz), while the remaining 21 occurred for net input power higher than optional levels. However, it is worth noting that the power level at which EMI events were observed was unlikely to be obtained by commercial 5G terminals. For pre-5G devices, PM/ICD manufactures typically indicate a safety distance of 15 cm that must be maintained to ensure the patient’s safety (according to what stated in the ISO 14117 standard). The results of our study demonstrate that such indication can also be considered valid for 5G terminals.

We specifically addressed several aspects that are known to play a role in inducing EMI, in particular, pulsed modulation, device sensitivity, dipole positioning, unipolar versus bipolar lead and sensing modality.

### 4.1. Pulsed Modulation

The principal RF interaction with implanted cardiac devices is spurious EMI signal generation through the undesired demodulation of high-amplitude RF signals on pacing leads and PM/ICD input stage. Spurious EMI signals, which are similar to the pulsating cardiac signal sensed by the cardiac device, are most likely to cause interactions. The 2 Hz RF modulation used in this study (475 ms OFF, 25 ms ON) is specified in ISO 14117. It can be considered a worst case condition, since it reproduces a rate and pulse width that simulates physiological signal characteristics and, as a result, lies within the band pass of the implantable device. This RF-pulsed signal is equivalent to a heart rate of 120 bpm. Additionally, we tested another modulation (330 ms OFF, 25 ms ON) to emulate an interfering signal with a frequency of 2.8 Hz, empirically chosen to mimic a ventricular tachycardia of 170 bpm. This latter modulation is not listed in the ISO 14117 tests, but it allowed us to observe if a modulated interfering signal with a timing that fits within the VT windows of the ICD is able to trigger a false VT detection. It is worth noting that typical communications which service signal modulations used by 5G systems are much less disturbing than both the modulations adopted in our measurements.

As expected, a continuous RF signal does not trigger EMI events; generally, it can only lead to some changes in the expected behavior of the devices that do not impact on patient safety (e.g., missing of a single pulse during pacing test or delivering of sporadic pulses during sensing test). Only a single PM showed the complete pacing inhibition, for power level higher than 15 W, when exposed to the continuous RF signal.

### 4.2. Device Sensitivity

We programmed the devices at the maximum sensitivity (minimum detection threshold) allowed by the manufacturer, that, in some cases, was even lower than the sensitivity value indicated in the standard. According to ISO 14117, for frequencies above 1 kHz, the least sensitive settings acceptable for compliance are 2.0 mV in the unipolar sensing mode and 0.3 mV in the bipolar sensing mode, or the sensitivity as shipped, whichever is the more sensitive. In most cases, the settings adopted in this study correspond to a more sensitive condition compared to what is prescribed by the standard. In one case, for a PM programmed in bipolar sensing, the minimum allowed sensitivity was 1 mV, greater than 0.3 mV.

As expected, EMI power thresholds are influenced by sensitivity, generally the lower the sensitivity, the lower the power threshold. However, the results observed in this study did not highlight a univocal correlation between the power levels at which EMI occurred and the device’s programmed sensitivity. Indeed, the device most susceptible to EMI was not the device programmed at the lowest sensitivity. On the other hand, the only device that was inhibited by the RF-sinusoidal signal had also the lowest sensitivity (0.1 mV).

### 4.3. Dipole Positioning

The presence of a preferential coupling between a specific dipole antenna orientation and lead connector, thus a preferential coupling more likely to generate an EMI event, is due to the fact that EM fields of hand-held transmitters affect implanted cardiac devices primarily through field-to-lead energy transfer at the connector [7]. In PMs with unipolar sensing, dipole position parallel to PM connector (position number 2) is more likely to cause EMI events because this specific setup configuration is a preferential coupling factor. On the contrary, PMs with bipolar sensing seem to be mostly affected by dipole perpendicular to connector (position number 1). However, for the latter, it is quite challenging to understand this coupling phenomenon since it is hardly predictable how tip and ring signals are treated and amplified by the input stage of the PM circuity. A similar consideration also applies to ICDs, for which there is not a clear pattern that indicates the presence of a preferential coupling positioning.

### 4.4. Unipolar versus Bipolar Lead

Devices that did not exhibit any EMI events when connected to bipolar lead did the same when connected to the unipolar one; similarly, devices that exhibit malfunctions when connected to bipolar lead did the same when connected to the unipolar one. In this latter case, the power threshold for EMI was lower for unipolar leads in 10 out of 12 tests.

### 4.5. Sensing Modality

The collected results for PMs do not give conclusive evidence. In most cases, programming the devices with bipolar sensing resulted in a higher immunity to EMI.

In one case, an EMI event was observed only in bipolar mode and not in unipolar mode. For one PM, the immunity threshold was higher in unipolar mode. At frequencies above 300 MHz, coupling mechanisms with tip and ring conductors and how the RF interfering signals are treated by input stage of PM are hardly predictable. Specific further assessment is needed to better investigate this aspect.

## 5. Future Plans and Potential Issues

This study considered only 5G frequency bands below 6 GHz (FR1). The FR2 band is expected not to pose significant risk to patients with AIMD, due to the increased device protection afforded by the attenuation of the enclosure and body tissue at microwave frequencies. However, further evaluation and ad hoc testing are still necessary to confirm such expected behavior.

In addition, future studies shall consider testing actual 5G signals (i.e., the actual 5G modulation and communication protocols), both for uplink (i.e., generated by the 5G mobile terminal) and downlink (i.e., generated by the 5G base station antenna).

## 6. Conclusions

The 5G technology is likely to become a pervasive technology and to significantly modify many aspects of our everyday life. Even if 5G systems do not need a higher power rating compared to pre-5G, they adopt new frequency bands that are not specifically considered by the current regulation framework and that may raise concerns on the potential interaction with AIMD. Today, the two main bands adopted by 5G mobile providers are 700 MHz and 3.6 GHz. The experimental measurements presented in this study reveal that RF hand-held transmitters operating in these two frequency bands do not pose additional risk compared to pre-5G bands and that the safety distance of 15 cm typically indicted by PM/ICD manufacturer is still able to guarantee the patient’s safety.

## Figures and Tables

**Table 1:** List of tested PMs with the specific models and programmed parameters.

Manufacturer	Model	Mode	Sensitivity (mV)	Base Pulse Rate (BPM)
Biotronik	Effecta DR	AAI	0.5	60
Medtronic	Sensia DR	VVI	1	60
Nayamed	NPX1 DR	VVI	1	60
Abbott (St. Jude)	Accent MRI	AAI	1	60
Microport (Sorin)	Kora 100 DR	AAI	0.4	60
Boston Scientific	Altrua	SSI	0.25	60

**Table 2:** List of tested ICDs with the specific models and programmed parameters.

Manufacturer	Model	Mode	Sensitivity (mV)	Base Pulse Rate (BPM)
Medtronic	Viva Quad S CRT-D	VVI	0.15	60
Biotronik	Inlexa 3 VR-T/DR-T	VVI	Auto	60
Abbott (St. Jude)	Fortify ST VR	VVI	Auto	60
Boston Scientific	Dynagen Mini	VVI	A: 0.25 V: 0.15	60

## Data Availability

Not applicable.
